# The Application of a Synthetic Biodegradable Temporizing Matrix in Extensive Burn Injury: A Unicenter Experience of 175 Cases

**DOI:** 10.3390/jcm13092661

**Published:** 2024-05-01

**Authors:** Christian Tapking, Adriana C. Panayi, Gabriel Hundeshagen, Benjamin F. Thomas, Emre Gazyakan, Bjoern Bliesener, Amir K. Bigdeli, Ulrich Kneser, Felix H. Vollbach

**Affiliations:** 1 Department of Hand, Plastic and Reconstructive Surgery, Microsurgery, Burn Center, BG Unfallklinik Ludwigshafen, Hand and Plastic Surgery, University of Heidelberg, 67071 Ludwigshafen, Germany; christian.tapking@googlemail.com (C.T.); adriana.panayi@icloud.com (A.C.P.); gabriel.hundeshagen@bgu-ludwigshafen.de (G.H.); benjamin.felix.thomas@gmail.com (B.F.T.); emre.gazyakan@bgu-ludwigshafen.de (E.G.); bjoern.bliesener@bgu-ludwigshafen.de (B.B.); amir.bigdeli@bgu-ludwigshafen.de (A.K.B.); ulrich.kneser@bgu-ludwigshafen.de (U.K.); 2 Division of Hand, Plastic and Aesthetic Surgery, Ludwig-Maximilians-University (LMU), 80539 Munich, Germany

**Keywords:** burns, efficacy, dermis substitute, Biodegradable Temporizing Matrix, Meek, BTM, risk factors

## Abstract

**Objectives**: Addressing extensive and deep burn wounds poses considerable challenges for both patients and surgeons. The NovoSorb^®^ Biodegradable Temporizing Matrix (BTM) emerged as a novel dermal substitute and has been subjected to evaluation in large burn wound cases, with a specific focus on identifying risk factors associated with suboptimal take rates. **Methods**: All patients with burn wounds greater than 10% body surface that underwent BTM treatment between March 2020 and November 2023 were eligible for inclusion. Univariate analyses and linear regression models were employed to discern risk factors and predictors influencing the take rates of both the BTM and split-thickness skin grafts (STSGs). **Results**: A total of 175 patients (mean age 56.2 ± 19.8 years, 70.3% male) were evaluated. The mean take rates of the BTM and STSGs were 82.0 ± 24.7% and 87.3 ± 19.0%, respectively. There were significant negative correlations between BTM take and the number of surgeries before BTM application (r = −0.19, *p* = 0.01), %TBSA and STSG take (r = −0.36, *p* = <0.001) and significant positive correlations between BTM and STSG take (r = 0.41, *p* ≤ 0.001) in addition to NPWT and STSG take (r = 0.21, *p* = 0.01). Multivariate regression analyses showed that a larger number of surgeries prior to BTM application (OR −3.41, 95% CI −6.82, −0.03, *p* = 0.04) was associated with poorer BTM take. Allograft treatment before BTM application (OR −14.7, 95% CI −23.0, −6.43,*p* = 0.01) and failed treatment with STSG before BTM application (OR −20.8, 95% CI −36.3, −5.23, *p* ≤ 0.01) were associated with poorer STSG take, whereas higher BTM take rates were associated with overall higher STSG take (OR −0.15, 95% 0.05, 0.26, *p* = 0.01). The Meek technique was used in 24 patients and showed similar take rates (BTM: 76.3 ± 28.0%, *p* = 0.22; STSG: 80.7 ± 21.1, *p* = 0.07). **Conclusions**: This study summarizes our findings on the application of a BTM in the context of large burn wounds. The results demonstrate that successful treatment can be achieved even in patients with extensive burns, resulting in satisfying take rates for both the BTM and STSG. The data underscore the importance of promptly applying a BTM to debrided wounds and indicate good results when using Meek.

## 1. Introduction

Severe burns are among the most impacting and intricate wounds, manifesting both short-term consequences, like infections and non-healing, as well as long-term effects, such as scarring and the loss of functionality [1]. The traditional approach for burns, and the current gold standard, involves excision and autologous skin grafting [2]. Nevertheless, this method may not be suitable for all wounds, particularly those with full-thickness defects that do not adequately support skin grafts [3]. Over the past few decades, a multitude of skin substitutes, ranging from biological to synthetic materials, have been developed, researched and clinically utilized [4].

The NovoSorb^®^ Biodegradable Temporizing Matrix (BTM), developed by PolyNovo Biomaterials Pty Ltd. in Port Melbourne, Australia, stands out as an innovative dermal substitute. This entirely synthetic product consists of a 2 mm thick biodegradable polyurethane open-cell foam, which is encased by a non-biodegradable sealing membrane. The open-cell matrix promotes vascular ingrowth and serves as a framework to support the formation of new dermal tissue, gradually breaking down over an 18-month timeframe. BTM therapy is a two-staged procedure, whereby a BTM is applied to the debrided wound bed and after 3 to 4 weeks, and/or when sufficient recapillarization is present, subsequently overlaid with split-thickness skin grafts (STSGs). Small vessels grow into the open cavities of the membrane and contribute to the formation of the new dermis [4,5], providing a sufficient wound bed for later skin grafts. The matrix itself undergoes hydrolytic degradation over a period of up to 18 months and provides stability for the neodermis. Following its approval, the BTM gained widespread popularity as a versatile tool for treating complex wounds stemming from various causes [6].

The effectiveness of a BTM in wounds of different etiologies has been reported in several pre-clinical and clinical studies, most of which have been case series or case reports [6,7,8]. In a prospective, multicenter trial, Lo et al. reported successful STSGs in 30 patients that suffered from full-thickness burns with BTM take rates of 88% and STSG take rates of 81% [9].

Recently, Betar et al. reported their experience on 55 patients with burns affecting more than 40% total body surface area (TBSA). It was found that the operation time was reduced significantly when a BTM was used compared to grafting alone [8].

More recently, several studies evaluating the benefits of a BTM in burns and other wounds were published [6,7,9,10,11]. Nonetheless, there is a dearth of studies focusing on the use of a BTM in extensive burn wounds. Consequently, the aim of this study was to assess the effectiveness of a BTM in the context of large burns and identify factors that could impact the treatment’s success.

## 2. Materials and Methods

Prior to starting the present single-center study, it was approved by the local ethics committee (Protocol number: 2023-16901). This study included all patients treated with a BTM who were admitted to our department between March 2020 and October 2023 and had at least 10% TBSA surgically treated.

### 2.1. Treatment Protocol at Our Institution

Surgical debridement was performed on all patients, irrespective of the burn mechanism. Based on the specific wound condition (e.g., size, infection, exposed functional structures), general condition of the patients and receptibility, a BTM was applied directly to all wounds or during a subsequent procedure a few days after excision. At our institution, a BTM is no replacement for autografts, since we see STSGs as the gold standard. Whenever the wound bed allows for it, an STSG is performed. When the wounds do not allow for an STSG because of full-thickness burns, a BTM is used. When the wounds showed no signs of infection and appeared visibly clean, the BTM was affixed using non-absorbable sutures or surgical staples. Following the manufacturer’s instructions on silver-containing dressings, Mepilex^®^ Ag (Mölnlycke Health Care, Gothenburg, Sweden) was applied above the BTM. Depending on the location and size of the wounds, negative pressure wound therapy (NPWT) was used when possible in order to provide stability for early mobilization. The first bedside dressing change was performed between five and seven days post-surgery. Additional interventions were performed in cases of a hematoma or infection affecting the BTM. Hematomas were manually cleared, ensuring hemostasis to restore contact between the BTM and the wound bed. Any pus beneath the BTM was cleansed, and affected areas were topically disinfected with Lavasept^®^ for an additional five to seven days, in conjunction with systemic antibiotic therapy. Wound swabs and clinical assessments were conducted every 48 h or at every dressing change to monitor progress. The removal of the sealing membrane occurred within 3–4 weeks, depending on factors such as the revascularization and success rate of the BTM, as well as the patient’s overall condition. The complete coverage of the defect was accomplished through an autologous STSG (split-thickness skin graft) or Meek skin grafts. Following grafting, the skin graft was protected with NPWT (negative pressure wound therapy), Mepilex^®^ Ag or foam dressings for a period of five days until the initial dressing change, during which the success rates were documented. The ratios of STSG application were 1:1, 1:1.5 and 1:2. When employing Meek skin grafts, in accordance with standard practice, the STSG was positioned on a 42 × 42 mm cork square with the dermal side facing downwards. It was then immersed in saline and processed through the Meek micrograft cutting device (Humeca, Borne, The Netherlands), dividing it into 196 squares. The epidermal side of the STSG was subsequently sprayed with adhesive dressing and allowed to dry for a minimum of eight minutes. Following this, the cork was pressed onto a pre-folded polyamide gauze with aluminum backing, and the plissé, along with all graft islands, was transferred to the gauze. After removing the cork, the plissé was stretched at all sides to achieve a skin expansion of up to 1:9, although typically, a skin expansion of between 1:3 and 1:5 is achieved at our institution. The aluminum supporting layer was then peeled off, resulting in an expanded gauze with 196 separated skin islands ready for grafting. Finally, the plissé was applied to the wound, with the graft side facing down, and secured with surgical staples. At our center, the pre-folded gauze could be covered with either absorbent foam dressing (e.g., Mepilex^®^ Ag from Mölnlycke Health Care, Gothenburg, Sweden) and/or negative pressure wound therapy (NPWT), followed by dry sterile gauzes and bandages, similar to the protocol after mesh grafting. Before applying Mepilex^®^ Ag, the plissés were covered with NPWT and/or fatty gauze. The plissé was carefully removed 10 days after transplantation, during which the success rate was evaluated.

### 2.2. Clinical Outcomes

We conducted a retrospective analysis of patient records for all cases of a burn size ≥10% TBSA treated with a BTM. The collected data parameters included sex, age, body mass index (kg/m^2^), the mechanism of burn injury, TBSA% affected and treated with a BTM, the number of surgeries performed before BTM application, the duration between patient admission and BTM application and the interval between BTM application and STSG transplantation. Additionally, we recorded the take rates of the BTM and STSG as percentages, wound swab results before BTM application (positive or negative), identification of exposed structures and the presence of comorbidities. The co-primary outcomes were the postoperative take rates of the BTM and STSG, determined clinically during surgical procedures or dressing changes and expressed as percentages. Regression models were employed to identify predictive factors for both outcome parameters.

### 2.3. Statistical Analysis

Microsoft Excel^®^ 2024 (Microsoft, Redmond, WA, USA) was employed for data collection and GraphPad Prism (GraphPad Software 9 for MacOS, La Jolla, CA, USA) for the analysis. Categorical variables are presented as numbers (*n*) and percentages (%), and continuous variables are presented as means with SD. Correlations between the main outcome of interest, i.e., the BTM percentage take, and various demographic and operative variables were assessed using Spearman’s R. A multivariate regression of all outcomes, i.e., BTM percentage take, STSG percentage take, LOHS and complication occurrence, was performed including the following variables as possible confounders: sex (female/male), TBSA (%), age (years), BMI (kg/m^2^), time to the BTM, %BTM, the number of surgeries before the BTM, treatment before the BTM, time from the BTM to STSG (days) and whether NPWT was applied on the STSG. For the LOHS, complications were also included in the multivariable analysis. The results of the multivariate analysis are presented as odds ratios (ORs) with 95% confidence intervals (CIs). P values less than 0.05 were considered significant.

## 3. Results

### 3.1. The General Characteristics of the Patient

We identified 175 patients with burns larger than 10% TBSA that were treated with a BTM. Of these, 52 (29.7%) were female. The patients’ mean age upon admission to the burn unit was 52.6 ± 19.8 years. The predominant mechanism of the burn injury was flame (*n* = 94, 53.7%), followed by scald (*n* = 36, 20.6%), electrical (*n* = 11, 6.3%) and other causes (*n* = 1, 0.6%). The most prevalent comorbidities included arterial hypertension (*n* = 45, 25.7%), peripheral artery disease (*n* = 31, 17.7%) and diabetes mellitus (*n* = 30, 17.1%). Detailed demographic information is provided in Table 1.

### 3.2. Information on Wounds and Treatment

The average wound size was 30.3 ± 19.8% of the total body surface area (TBSA), with 9.7 ± 12.1% of the TBSA being treated with a BTM. The mean duration from injury to BTM transplantation was 14.3 ± 31.9 days and from BTM to STSG transplantation was 28.4 ± 10.1 days. Most of the patients (*n* = 139, 79.4%) did not receive any transplantation before the application of the BTM, while 24 patients (13.7%) underwent a prior allograft, and five patients (2.9%) received autograft transplantation. Before the application of the BTM, positive wound swabs from clinically clean wound beds were detected in 107 cases (61.1%).

The average BTM take rate was 82.0 ± 24.7%, and the take rates of the STSG after BTM delamination were 87.3 ± 19.0%. The most common complications of the BTM included infection (*n* = 36, 20.6%) and hematomas (*n* = 7, 4.0%), which were treated with intensive bedside disinfection (as described above) in 32 cases (18.3%) and led to BTM removal in 15 patients (8.6%). Details regarding wound characteristics and treatment are provided in Table 2.

### 3.3. Pooled Estimates of Entered Covariates

When examining correlations between various parameters and the BTM take, it was revealed that the number of previous surgeries exhibited a negative correlation (r = −0.19, *p* = 0.01). No significant correlations were observed for variables such as sex, the etiology of the respective wound, affected size, the presence of infections or treatment with NPWT (Table 3). In terms of the STSG take, burn size demonstrated a negative correlation (r = −0.36, *p* ≤ 0.001), while BTM take (r = 0.41, *p* ≤ 0.001) and NPWT treatment (r = 0.21, *p* = 0.01) exhibited positive correlations. No significant correlations were found for age, sex or the time from the BTM to STSG (Table 3). Pooled odds ratio (OR) estimates for all evaluated covariates linked to both co-primary endpoints are shown in Table 4. Patients with a larger number of surgeries before BTM application showed a negative effect associated with lower BTM take rates (estimate −3.41, 95% CI −6.82, −0.03, *p* = 0.04).

In terms of STSG take rates following BTM delamination, the most notable positive correlation was identified in instances where the BTM take was higher (estimate 0.15, 95% CI 0.05, 0.26, *p* = 0.01). Conversely, a negative impact was noted in patients who had undergone prior allograft transplantation, linked with lower STSG take rates (estimate −14.7, 95% CI 23.0, −6.43, *p* = 0.01) and prior STSGs (estimate −20.8, −36.3, −5.23, *p* < 0.01). No significant association was identified for the length of stay. Male patients were found to have fewer complications (estimate −0.15, 95% CI −0.30, −0.01, *p* = 0.04, Table 5).

### 3.4. Subgroup Analysis of Meek Patients

There were 24 patients who were treated using the Meek technique and presented with a mean burn size of 47.9 ± 19.0%. The average time from injury to BTM application was 6.0 ± 6.3 days and from BTM to Meek 31.3 ± 13.2 days.

The mean take rate of the BTM and STSG was 76.3 ± 28.0% and 80.7 ± 21.1%, respectively, which did not differ from the take rates of patients not treated with the Meek technique (BTM: *p* = 0.22; STSG: *p* = 0.07). All patients had some kind of complication of the BTM such as hematomas or infection. These complications were mild in most cases but led to the (partial) removal of the BTM in four cases (16.7%).

## 4. Discussion

Large burn wounds are complex to treat, both surgically and in intensive care. In the present study, 175 patients with burns greater than 10% TBSA and a mean of 30% TBSA burned were treated with a BTM covering 10% TBSA on average and subsequently covered with an STSG. In general, the take rates of the BTM (82%) and STSG (87%) were high. In a subgroup of 25 patients, the Meek technique was used to cover the BTM (BTM take 76% and Meek take 81%). One significant predictor for the STSG take rate was found to be the BTM take rate, showing a strong positive correlation. It was also shown that a larger number of surgeries prior to BTM application was a negative predictor for BTM take.

The preferred method for managing severe burns typically entails the prompt removal of burn eschar, followed by the application of autologous split-thickness meshed skin grafts for coverage [2]. This method is especially favored. However, challenges arise in cases of particularly extensive and deep burn wounds, where the limited availability of donor sites poses a hurdle to the utility of this technique. Previous studies have indicated that delayed wound healing and closure are linked to the development of hypertrophic scarring [12,13].

Often, the successful treatment of burn wounds cannot be achieved using only one technique. Due to varying burn depths, a mixture of dermal substitutes such as a BTM and regular STSG is indicated. In the present study, the patients presented with burns of about 30% TBSA. A mean of 10% were treated with a BTM, whereas the rest were immediately covered with STSGs. The high take rates of the BTM show that it can be used safely and as a good adjuvant when the wounds are not appropriate for direct skin grafting in patients with a large TBSA affected. Other advantages, especially in large burns, are the fast and easy application and the fact that surgery times tended to be shorter when the BTM was used, while the intervals between dressing changes were longer. Therefore, the patients can recover from intensive care issues and can be mobilized earlier. Extensive and full-thickness wounds, whether resulting from burns or other causes, are frequently susceptible to infection. They often necessitate multiple corrective surgeries and are characterized by less favorable long-term outcomes in terms of functionality, appearance and scarring [14,15,16].

Prior to the introduction of the BTM to our institution, we used allografts for temporary coverage to prepare the wound for STSGs or skin substitutes. As the use of the BTM became a more standard practice, the importance of early BTM application on clean wounds and its ability to reduce the number of revisions, infections and BTM takedown became apparent. The fact that in our analysis, the STSG take rates were lower when allografts or STSGs were used before the BTM reflects this experience. Also, a larger number of surgeries before BTM was found to be a negative predictor for BTM take. Utilizing allografts is a prevalent approach in addressing burn injuries, particularly in scenarios where there are inadequate donor sites to cover all affected areas simultaneously or when patients are in unstable conditions, requiring expedited surgical intervention. Subsequent procedures often entail removing the allograft and replacing it with either skin substitutes or autologous skin grafts if the wound bed is deemed appropriate for STSGs. However, allografts present drawbacks, such as a heightened risk of infections, the necessity for frequent dressing changes and limited availability [17]. Additionally, larger wounds covered with allografts have been reported to be particularly susceptible to infections, often requiring more surgeries [18]. This observation might partially elucidate the findings of the present study, where prior allografts were recognized as a detrimental factor for STSG take rates, as contaminated wounds also exerted a negative influence on these parameters.

As anticipated, there was a positive correlation between high BTM take rates and high STSG take rates. These findings align with a prospective study conducted by Lo et al., analyzing 30 burn patients, where they reported a BTM take of 88.6% and an STSG take of 81.9% [9]. Notably, they identified that prior allograft transplantation was a parameter exerting a negative influence on STSG take rates.

Recently, Austin et al. investigated the influence of NPWT on the BTM take rate and reported a significantly higher integration rate when the BTM was covered with NPWT (93.8% vs. 58.3% without NPWT). At our institution, NPWT is commonly used, and a similar pattern was identified. However, NPWT application is not always feasible, especially with very large wounds, in which case we used Mepilex Ag as a foam dressing cover for the BTM for approximately five weeks. Recently, Schlottmann et al. documented their findings based on two patients with full-thickness wounds arising from burns, trauma and various etiologies. Their study suggests that the BTM is a dependable and safe reconstructive option, particularly in cases involving patients with multiple comorbidities and infected wounds. [10].

The typical protocol involves leaving the BTM undisturbed for a period of 3–4 weeks, occasionally extending the duration to ensure the adequate integration of the BTM layers with the wound bed. Consequently, infections with pus can be detected clinically beneath the BTM during this period. Our approach involves the manual cleaning of the wounds and pus evacuation under local anesthesia (with the potential need for short general anesthesia in large burn wounds) to restore contact between the BTM and the wound bed, thereby averting the need for BTM removal. Despite our consistent practice of applying the BTM solely to wounds that are clinically clean and well debrided, this study underscores the significance of factoring in objective measures like wound swabs prior to BTM application. Interestingly, positive wound swabs did not emerge as a risk factor for inferior STSG take rates in this study, given that the STSG was exclusively conducted after the wound had already been adequately covered with the BTM. It is important to note that positive wound swabs indicate a contamination but do not necessarily mean that there is a clinically relevant infection. In this study, the BTM was applied around 14 days after injury, and the STSG was performed approximately four weeks after BTM application. While this study includes patients taken from over a period of 3.5 years, we learned over time that early application is beneficial for patients in many ways, such as the prevention of infection, early mobilization, the lower frequency of dressing changes or recovery from intensive care issues. Also, the STSG is time-flexible and does not have to be performed strictly after four weeks. In general, one can “buy time” by early excision and early BTM application.

In critical cases, the BTM serves as a potential lifesaving option or a viable alternative to amputations, offering advantages such as reduced resource requirements and shorter operation times compared to procedures involving free flaps or extensive autologous skin grafting. This is particularly beneficial for patients who are not suitable candidates for immediate excision and autologous grafting [11]. The BTM offers a shield against potential infections, a risk more common with temporary allograft coverage. Unlike allografts, the BTM negates the need for frequent dressing changes, enabling early physical therapy. Moreover, postponing the second surgery until patient stabilization or adequate donor sites for STSGs are available is feasible. In major burn cases, the BTM negates the requirement for frequent allograft changes during the 3–4-week interim until it is healed and ready for grafting. However, healing may be prolonged in patients with diabetes or those with vascular interventions [19,20].

It is important to note that a key finding over the course from the introduction of the product to today was the timing of BTM application. As we did not have experience in the beginning, the wounds were excised and then temporarily covered using an allograft or NPWT, and later on, the BTM was applied. Over time, we learned that this frequently causes the contamination of the wound, especially when using an allograft. Therefore, infection rates of the BTM were higher, and take rates were lower. Today, in most cases, we use the BTM in the very beginning, when the wound is excised and STSG is not possible. This way, we can make sure to apply the BTM on a clean wound. Also, early BTM application may avoid numerous dressing and allograft changes.

When donor sites are limited, a technique such as Meek can be used to cover large wounds. Using the Meek technique can be a fast and safe option, with reported take rates of up to 90% [21,22]. The take rates often differ, especially in terms of the used ratio and the burn size. Several studies, including a recent one from our institution, showed take rates between 75 and 80% [23,24]. This is in line with our study, in which 81% take rates were seen. This is the first time that Meek on a BTM is reported. There was no difference between patients treated with STSG or Meek after BTM application. Most likely, the success on a regular wound bed is different compared to using a BTM. However, this indicates that Meek on a BTM can be a promising and safe option for surgeons when treating patients with massive burns.

### Limitations

The simple design of this study with retrospective analyses and single-center data is a limitation per se. However, over the last few years, we were able to treat a large number of patients, which were included in the present study. Similar to any other new technique or device, there is also a learning curve with BTM use. In every institution, there might be slight adjustments to the manufacturer’s recommendations for BTM application based on growing experience and caseload. For example, in the beginning, we waited longer periods prior to the application of the BTM compared to later cases. This may somehow influence the outcomes and should be noted. The primary outcomes, STSG and BTM take rates, were assessed through both clinical evaluation and subjective judgment by the overseeing surgeons. However, it is essential to note that the patients were treated by highly experienced surgeons with extensive expertise, ensuring consistency in the subjective parameters used for evaluating take rates. Additionally, it is important to acknowledge that, due to the inherent nature of statistical methods, potential confounders or co-linearity may exist within the models employed.

## 5. Conclusions

In this study, we shared our experiences with a BTM in large burn wounds. Here, we were able to demonstrate that successful treatment can also be performed in patients with large burns leading to satisfactory take rates for the BTM and STSG. The data indicate that the BTM should be applied as soon as possible on clean wounds. Using the BTM promptly in patients with large burns can improve recovery from intensive care issues, favor the early mobilization of the patient, extend the intervals of dressing changes compared to allografts and allow for more flexibility in terms of further wound coverage timing. It has also been shown that the BTM can safely be covered using the Meek technique. This extends the surgical armamentarium of surgeons, especially in massive burns, where donor sites are scarce. This study analyzes the short-term results of the BTM in burn patients. Long-term results in terms of functionality, scarring and the quality of life require further investigation.

## Figures and Tables

**Table 1:** Patient demographics and wound characteristics. Reported as *n* (%), unless otherwise stated.

	Total (*n* = 175) (*n* = 73)
Age in years, M (SD)	52.6 (19.8)
BMI in kg/m^2^, M (SD)	27.6 (5.9)
Sex	
Male	123 (70.3)
Female	52 (29.7)
TBSA%, M (SD)	30.3 (19.8)
TBSA% third degree, M (SD)	11.6 (15.2)
ABSI score, M% (SD)	7.1 (2.3)
Cause of wound	
Burn	149 (85.1)
Contact	6 (3.4)
Electrical	11 (6.3)
Flame	94 (53.7)
Scald	36 (20.6)
Other	1 (0.6)
Chemical	2 (1.1)
Chronic wound	5 (2.9)
Infection	7 (4.0)
Trauma	7 (4.0)
Tumor	3 (1.7)
Infected wound (positive swab)	107 (61.1)
Comorbidities	
Diabetes	30 (17.1)
Peripheral Arterial Disease	31 (17.7)
Coronary Artery Disease	25 (14.3)
Hypertension	45 (25.7)
History of Smoking	29 (16.6)

M, mean; SD, standard deviation; n, number; BMI, body mass index; TBSA, total body surface area; ABSI, Abbreviated Burn Severity Index.

**Table 2:** Overview of operative management and outcomes. Reported as *n* (%), unless otherwise stated.

Operative Management	Total (*n* = 175) (*n* = 73)
Time to BTM in days, M (SD)	14.3 (31.9)
Time from BTM to STSG in days, M (SD)	28.4 (10.1)
Number of surgeries before BTM, M (SD)	1.0 (1.4)
Treatment before BTM	
None/NPWT	139 (79.4)
Allograft	24 (13.7)
STSG	5 (2.9)
Meek on Biodegradable Temporizing Matrix	24 (13.7)
% BTM, M (SD)	9.7 (12.1)
Size BTM in cm^2^, M (SD)	1785.6 (2361.6)
STSG Ratio	
1:1	19 (10.9)
1:1.5	105 (60.0)
1:2	4 (2.3)
1:3	3 (1.7)
Dressing on BTM	
Mepilex^®^ absorbent foam dressing	79 (45.1)
Mepilex^®^ absorbent foam dressing with NPWT	52 (29.7)
NPWT with simple foam dressing	39 (22.3)
Outcomes	
BTM take %, M (SD)	82.0 (24.7)
STSG take %, M (SD)	87.3 (19.0)
Number of surgeries after STSG, M (SD)	1.6 (0.8)
Type of surgery after STSG	
BTM	1 (0.6)
Further STSG	10 (5.7)
Flap	10 (5.7)
Other	5 (2.9)
LOHS in days, M (SD)	55.1 (47.0)
Mortality	16 (9.1)
Complication	47 (26.9)
Hematoma under BTM	7 (4.0)
Infection under BTM	36 (20.6)
Intensive bedside disinfection	32 (18.3)
BTM removal	15 (8.6)
Reoperation	17 (9.7)

M, mean; SD, standard deviation; n, number; BTM, Biodegradable Temporizing Matrix; NPWT, negative pressure wound therapy; STSG, split-thickness skin graft; LOHS, length of hospital stay.

**Table 3:** An assessment of the correlation between BTM take (%) and the listed variables.

**BTM Take (%)**	**Spearman R**	**95% CI**	***p*-Value**
Age	−0.14	−0.29–0.01	0.07
Sex, Male	−0.12	−0.27–0.04	0.13
BMI (kg/m^2^)	0.02	−0.13–0.18	0.78
TBSA (%)	−0.15	−0.30–0.01	0.06
Time to BTM (days)	0.04	−0.11–0.20	0.57
% BTM	−0.08	−0.23–0.08	0.30
Number of surgeries before BTM	−0.19	−0.34–−0.04	0.01
NPWT application	0.13	−0.02–0.28	0.09
Infected wound (positive swab)	0.11	−0.04–0.27	0.14
**STSG Take (%)**	**Spearman R**	**95% CI**	***p*-Value**
Age	−0.04	−0.20–0.13	0.64
Sex, Male	−0.09	−0.25–0.08	0.28
BMI (kg/m^2^)	0.14	−0.03–0.30	0.09
TBSA (%)	−0.36	−0.50–−0.21	<0.0001
Time from BTM to STSG (days)	−0.08	−0.24–0.08	0.32
BTM take (%)	0.41	0.27–0.54	<0.0001
NPWT application	0.21	0.05–0.36	0.01
Infected wound (positive swab)	0.02	−0.14–0.19	0.76

**Table 4:** A multivariate assessment of the outcomes. The variables included in the analysis are age (years), sex (female/male), BMI (kg/m^2^), TBSA (%), time to the BTM, %BTM, the number of surgeries before the BTM, treatment before the BTM, time from the BTM to STSG (days) and whether NPWT was applied on the STSG. LOHS complications were included in the multivariable analysis.

Outcomes	Estimate	95% CI	*p*-Value
BTM take (%)			
Age	−0.04	−0.25 to 0.17	0.72
Sex—Male	−1.14	−10.1 to 7.84	0.80
BMI (kg/m^2^)	−0.04	−0.72 to 0.63	0.90
TBSA (%)	−0.11	−0.36 to 0.15	0.40
Time to BTM (days)	−0.03	−0.16 to 0.10	0.66
Number of Surgeries before BTM	−3.41	−6.82 to −0.03	0.04
Treatment before BTM—Allograft	16.2	−14.1 to 12.8	0.91
Treatment before BTM—STSG	−0.70	−8.88 to 41.4	0.20
STSG take (%)			
Age	−0.06	−0.20 to 0.07	0.37
Sex—Male	1.08	−4.63 to 6.78	0.71
BMI (kg/m^2^)	−0.27	−0.73 to 0.18	0.24
TBSA (%)	−0.02	−0.21 to 0.16	0.81
Time to BTM (days)	0.02	−0.15 to 0.20	0.78
Meek prior to BTM	−6.40	−14.9 to 2.07	0.14
BTM take (%)	0.15	0.05 to 0.26	0.01
Time from BTM to STSG (days)	−0.23	−0.49 to 0.03	0.08
Treatment before BTM—Allograft	−14.7	−23.0 to −6.43	0.01
Treatment before BTM—STSG	−20.8	−36.3 to −5.23	<0.01
LOHS			
Age	0.02	−0.35 to 0.38	0.92
Sex—Male	−1.61	−17.4 to 14.2	0.84
BMI (kg/m^2^)	−1.08	−2.33 to −0.17	0.09
% BTM	0.11	−0.50 to 0.72	0.71
Complications	−13.7	−30.1 to 2.79	0.10
Complications			
Age	0.00	0.00 to 0.00	0.53
Sex—Male	−0.15	−0.30 to −0.01	0.04
BMI (kg/m^2^)	0.00	−0.01 to 0.01	0.83
% BTM	−0.01	−0.01 to 0.00	0.07
Time to BTM (days)	0.00	0.00 to 0.00	0.77
Infected wound (positive swab)	0.00	−0.14 to 0.14	0.99

n, number; TBSA, total burn surface area; NPWT, negative pressure wound therapy; STSG, split-thickness skin graft; BTM, Biodegradable Temporizing Matrix.

**Table 5:** Overview of operative management and outcomes in patients receiving Meek on Biodegradable Temporizing Matrix. Reported as *n* (%), unless otherwise stated.

Operative Management	Total (*n* = 24) (*n* = 73)
TBSA (%), M (SD)	47.9 (19.0)
Time to BTM in days, M (SD)	6.0 (6.3)
Time from BTM to STSG in days, M (SD)	31.3 (13.2)
Number of surgeries before BTM, M (SD)	0.9 (1.5)
Outcomes	
BTM take %, M (SD)	76.3 (28.0)
STSG take %, M (SD)	80.7 (21.1)
Number of surgeries after STSG, M (SD)	1.6 (0.8)
Further STSG	5 (20.8)
LOHS in days, M (SD)	67.6 (27.2)
Mortality	4 (16.7)
Complication	24 (100.0)
Hematoma under BTM	4 (16.7)
Infection under BTM	8 (33.3)
Intensive bedside disinfection	8 (33.3)
BTM removal	4 (16.7)
Reoperation	7 (29.2)

M, mean; SD, standard deviation; n, number; BTM, Biodegradable Temporizing Matrix; STSG, split-thickness skin graft; LOHS, length of hospital stay.

## Data Availability

Data is available upon reasonable request to the corresponding author.
